# Prevalence and Risk Factors of Soil-Transmitted Helminths Among Students in Fogera District, Northwest Ethiopia

**DOI:** 10.1155/ghe3/3259544

**Published:** 2025-10-31

**Authors:** Miseganaw Sisay, Tadesse Hailu, Woyneshet Gelaye, Alemtsehay Kolech, Solomon Alebele, Destaw Damtie

**Affiliations:** ^1^Department of Biology, College of Natural and Computational Science, Debre Tabor University, Debre Tabor, Ethiopia; ^2^Department of Medical Laboratory Science, College of Medicine and Health Sciences, Bahir Dar University, Bahir Dar, Ethiopia; ^3^Department of Biology, College of Science, Bahir Dar University, Bahir Dar, Ethiopia; ^4^Department of Biology, College of Science, Sine-Hosaena Research Center, Bahir Dar University, Bahir Dar, Ethiopia

**Keywords:** *A. lumbricoides*, fogera, hookworm, prevalence, schoolchildren

## Abstract

**Background:**

Soil-transmitted helminth (STH) infection is a widespread problem globally, particularly in rural areas. In Ethiopia, the prevalence of STH infection is high. The prevalence of *Ascaris lumbricoides* and hookworm infections has not diminished in spite of the nation's strict STH prevention and control measures for decants. Additionally, variables linked to the high prevalence of *A. lumbricoides* and hookworm infections are not adequately addressed.

**Objective:**

This study aimed to determine the prevalence and risk factors of STHs among students in the Fogera district, northwest Ethiopia.

**Method:**

A cross-sectional study design was employed in two primary schools in the Fogera district in February–March 2023. The stool samples were collected from the students and examined using saline wet mount and double Kato–Katz technique. Data were entered and analyzed using SPSS Version 25. Descriptive statistics was used to compute the prevalence of STHs. Factors associated with hookworm and *A. lumbricoides* infections were analyzed by logistic regression. Variables with *p* < 0.05 in the multivariate logistic regression were considered significant.

**Results:**

Among 463 schoolchildren, totally, the prevalence of STHs was 25.3%. The prevalence of *A. lumbricoides* and hookworm infections was 55 (11.9%) and 62 (13.4%), respectively. Mothers' lack of education (AOR = 24.99; 95% CI = 7.05–88.67; *p* < 0.001), fathers' lack of education (AOR = 3.03; 95% CI = 1.18–7.7; *p* = 0.021), school latrine nonusage at school (AOR = 4.53; 95% CI = 1.89–10.95; *p* < 0.001), untrimmed fingernails (AOR = 7.31; 95% CI = 2.57–20.81; *p* < 0.001), no handwashing after toilet use (AOR = 14.87; 95% CI = 3.97–55.75; *p* < 0.001), no handwashing before eating (AOR = 30.05; 95% CI = 11.05–81.74; *p* < 0.001), the absence of handwashing facility at home (AOR = 5.86; 95% CI = 2.19–15- 64; *p* < 0.001), and irregular wearing of shoes (AOR = 18.59; 95% CI = 6.89–50.13; *p* < 0.001) were significantly associated with STH infection.

**Conclusion:**

The prevalence of *A. lumbricoides* was significant. Poor implementation of water, sanitation, and hygiene was a risk factor for *A. lumbricoides* and hookworm infection. Hence, health education on the transmission of STHs should be advocated to parents and schoolchildren.

## 1. Introduction

Soil-transmitted helminths (STHs) refer to the intestinal worms infecting humans that are transmitted through contaminated soil (“helminth” means parasitic worm): *Ascaris lumbricoides* (sometimes called just “*Ascaris*“), whipworm (*Trichuris trichiura*), and hookworm (*Ancylostoma duodenale* and *Necator americanus*) [1]. STH infections are among the most common infections worldwide and affect the poorest and most deprived communities. They are transmitted by eggs present in human feces, which in turn contaminate soil in areas where sanitation is poor [2]. Intestinal worms produce a wide range of symptoms including intestinal manifestations (diarrhea and abdominal pain), general malaise, and weakness. Hookworms cause chronic intestinal blood loss that results in anemia. STHs are transmitted by eggs that are passed in the feces of infected people. Adult worms live in the intestine where they produce thousands of eggs each day [3].

STH infections are grouped by the World Health Organization (WHO) as neglected tropical diseases [2]. Over 1.5 billion people are infected with STH infections worldwide or 24% of the world's population. Infections are widely distributed in tropical and subtropical areas, with the greatest numbers occurring in sub-Saharan Africa, the United States, China, and East Asia [3]. From STH, *A. lumbricoides* and *T. trichiura* are the most common nematode parasites of humans in Ethiopia. However, in hookworm, *N. americanus* is more prevalent in Ethiopia than *An. duodenale*, species of hookworm [4].

The poorest and most marginalized groups are primarily affected by STH infections, which are among the most prevalent infections globally [3]. Over 1.5 billion individuals globally, or 24% of the global population, suffer from STH diseases [3]. Their infections are prevalent in warm, moist climates with poor sanitation and hygiene [1]. They are the most common worldwide, affecting impoverished communities [1, 5]. The transmission occurs through eggs present in human feces, contaminating soil in areas with inadequate sanitation. Intestinal worms cause various symptoms, including diarrhea, abdominal pain, general malaise, and weakness [3]. Tropical and subtropical regions are rife with infections; sub-Saharan Africa, the United States, China, and East Asia have the highest rates of infection. More than 568 million school-age children and over 267 million preschool-age children who reside in locations where these parasites are heavily spread due to unwashed hands after defecation, unwashed hands after playing with soil, being barefoot, unavailable latrines, not dewormed regularly, and limited awareness have a high prevalence of infections [3].

Ethiopia ranks 13^th^ among more than 40 African countries in terms of the prevalence of each group of illnesses [6]. STH is still highly prevalent in many areas of Ethiopia particularly among schoolchildren [7]. Children from rural communities have a higher prevalence of STHs compared to those from urban communities. The prevalence of STH in Ethiopia is 33%, with *A. lumbricoides* being the most common among children [8]. Parasitic helminth infections rank as the second most common cause of outpatient morbidity in Ethiopia due to factors such as poor toilet coverage and low-quality drinking water [9]. The pooled prevalence estimates for *A. lumbricoides* and hookworm in Ethiopia are 17.63% and 12.35%, respectively [10].

The intensity of *A. lumbricoides* and hookworm varies by location in Ethiopia. For example, the study conducted in Tachgayint indicated a light intensity [11], and the study conducted in Jimma indicated that the intensity of these infections is moderate. The number of worms in an individual's body determines the intensity of *A. lumbricoides* and hookworm infections. This is usually determined by counting the number of eggs in a stool sample. A larger egg count implies a more severe illness [1].

The identification of risk factors associated with STHs may vary across different locations, making it essential for policymakers to have this information to develop targeted preventive strategies [12]. Factors such as warm climates, adequate moisture, poor personal and environmental hygiene, lack of sanitation and education, walking barefoot, and poor health or nutrition status can increase the risk of infection [13]. In Ethiopia, poor living standards, inadequate sanitation, and limited knowledge about basic health promotion contribute to the widespread distribution of intestinal helminths [14]. Helminthiasis ranks as the second most common cause of outpatient morbidity in the country, following malaria [15]. Extensive research conducted in Ethiopia indicates a significant prevalence of intestinal parasite infections. Children, who both spread and suffer from these diseases, are most affected by parasite infections such as *A. lumbricoides* and hookworm [16].

Despite control efforts, the infections remain prevalent and show no significant decline [17]. The Fogera district, as reported in its annual health report, experiences a higher prevalence compared to other districts in the South Gondar Zone administration, especially schoolchildren [18, 19]. The prevalence of infections among schoolchildren in the research region is currently unknown. Factors such as open defecation, limited toilet coverage, lack of access to clean drinking water and sanitation facilities, and unhygienic practices such as walking barefoot and consuming unwashed raw fruits and vegetables from the market have been observed. Therefore, this study aims to fill this knowledge gap by determining the prevalence and associated risk factors of soil-transmitted infections among primary school students in the Fogera district, northwest Ethiopia.

## 2. Materials and Methods

### 2.1. Description of the Study Area

**Figure 1:**
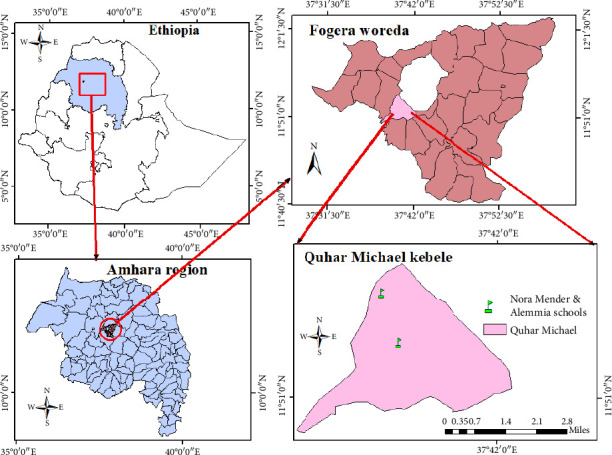
Map of the study area.

The study was conducted in two primary schools at the Fogera district, Amhara regional state, northwest Ethiopia (Figure 1). The schools are situated 4 km away from Woreta town, the capital city of the district, 44 km from Debre Tabor (the capital of South Gondar Zone), 57 km from Bahir Dar (the capital of Amhara regional state), and 612 km from Addis Abeba (the capital city of Ethiopia). The schools have a total of 1025 pupils, with 801 in Nora Mender and 224 in Alemmia. The majority of the population are farmers engaged in settled mixed agriculture, focusing on crops such as rice, onions, and maize, using both irrigation practices and the summer season. Geographically, the area is located at latitude 180 42′ 34″N and longitude 310 16′ 84″E, with an altitude of 1847 m above sea level. The mean annual rainfall in the study area is approximately 1280 mm, and the mean annual minimum and maximum temperatures are 11.9°C and 28.84°C, respectively, with a mean temperature of 20.37°C (Woreta Administrative Office, 2020).

### 2.2. Study Design

A cross-sectional study design was employed to determine the prevalence and associated risk factors of *A. lumbricoides* and hookworm infection among students in Fogera.

### 2.3. Study Population

Schoolchildren were selected as the study population because school-age children typically have the highest intensity of worm infection. Hence, the study population consisted of 463 (216 males and 247 females) schoolchildren (6–14 years) enrolled in February–March 2023 in Fogera.

### 2.4. Sampling Techniques and Sample Size Determination

The participants were selected by systematic random sampling techniques to ensure the inclusion of STH infections. So, the *K*th interval was determined by dividing the total sample size of 463 by the total study population of 1025 (from 224, 115 were selected in Alemmia school, and out of 801, 348 were selected from Nora Mender school), resulting in a *K*th interval value of ∼2. Next, the first study participants were chosen using a simple random sampling technique, and the second study participants were selected through a lottery system. Subsequently, every *K*th (second) interval participant was selected until the total sample size of 463 was achieved.

The required sample was calculated using the following formula [17]:t

(1) $$\begin{array}{c} n=\frac{z2\;P\left(1-P\right)}{D2\;\left(0.05\right)}. \end{array}$$

There is no study conducted in the study area about prevalence and associated risk factors of *A. lumbricoides* and hookworm infections among schoolchildren. Hence, based on the above formula, the sample size (*n*) would be 384 due to the *p* value of 50%, which is the lack of the study on the prevalence in the study area. To account for a 21% nonresponse rate, an additional 21% of the calculated sample size was added. Therefore, the total sample size was 384 + 384 ∗ 0.21 = 463. Thus, a total of 463 students (247 females and 216 males) were selected from the primary schools of the Fogera district.

The intensity of infections was determined by using the threshold level reported by WHO [20] (Table 1).

### 2.5. Eligibility Criteria

#### 2.5.1. Inclusion Criteria

This study included students who tested positive for STH infections from primary schools that were highly vulnerable to STH due to the presence of lowland and suitable soil types at the Fogera district. The age range of the students was between 6 and 14 years. Inclusion criteria required that the parents/guardians of the children agreed to their participation in the study, students tested positive for STHs, and students aged 6–14 without recent deworming.

#### 2.5.2. Exclusion Criteria

Students who tested negative for parasitic infections, were in the age group above/below the required, were with diarrheic stool, with anti-helminthic drugs, or had previously consumed antihelminthic drugs, or whose parents/guardians did not agree to their participation in the study were excluded from the present study.

### 2.6. Data Collection Method

#### 2.6.1. Questionnaire Survey

The questionnaire was initially written in English. After that, it was translated into Amharic, the working language, so that study participants could provide sociodemographic information and information about risk factors for STH infection. Throughout the data collection process, each study participant received comprehensive explanations regarding the purpose of the study, and sociodemographic data and risk factors of the study participants who are unable to read and write were collected by the data collector. Furthermore, to ensure the anonymity and confidentiality of the collected data, all 463 participants were assigned a unique identification number.

#### 2.6.2. Stool Collection Process and Parasitological Examination

The authors went to the study site, which is the two primary schools of Fogera, for the collection of the stool sample for parasitological examination. The students were provided with instructions on how to collect stool samples. Labeled stool collection cups with applicator sticks were distributed to each child, and they were instructed to provide close to 2 g of fresh stool specimens. The students went to the toilet of the school to bring the stool sample for the lab examination. The collected stool samples from the students were transported to Woreta Health Centre in an icebox with an ice pack for laboratory processing to prevent the parasite from being damaged by environmental factors like temperature. To examine the stool samples for the presence or absence of STHs, direct wet mount microscopy and two slides of the Kato–Katz technique were utilized. The examination was performed by two medical laboratory technologists using a compound light microscope.

Data quality was meticulously maintained through a series of precise measures. To begin with, laboratory personnel underwent comprehensive training on various aspects, including stool sample collection, processing, diagnosis, and reporting. Similarly, data collectors were trained on using questionnaires, and these questionnaires were pretested before actual data collection.

Additionally, thorough checks were conducted to ensure the integrity of the collected data. The filled questionnaires were meticulously examined to ensure completeness, and the stool cups were appropriately labeled with unique serial numbers. Moreover, the samples were promptly transported to the nearby health institution without delay.

Stringent adherence to standard operating procedures was maintained throughout the entire process, emphasizing the accuracy of the test results. Regular supervision by the principal investigator ensured consistent quality control and oversight. To mitigate any potential observer bias, stool slides were examined independently by experienced laboratory personnel. Their observations were recorded on separate sheets for later comparison. In cases where discrepancies emerged, the principal investigator personally reviewed and verified the results. Overall, the quality assurance procedures were implemented across the pre-analytical, analytical, and postanalytical stages. This comprehensive approach ensured that the data collected and analyzed maintained a high standard of accuracy and reliability.

### 2.7. Variables of the Study

#### 2.7.1. Independent Variables

The independent variables in this study encompassed a range of sociodemographic characteristics (age, sex, parental education, name of school), environmental characteristics (drinking water source), behavioral and personal characteristics (frequent sucking of fingernails, lack of handwashing before eating and after toilet use, and eating raw vegetables or fruits), and socioeconomic characteristics (not wearing shoes and washing facility at home).

#### 2.7.2. Dependent Variables

The prevalence of STH infection was the dependent variable of this study.

### 2.8. Method of Data Analysis

The data were entered into Excel 2016 and analyzed using Statistical Package for the Social Sciences (SPSS) Version 25 software for the prevalence and risk factors. The prevalence of STHs was calculated as the percentage of children who tested positive for these species out of the total number of children with complete data. The association and strength of association between risk factors and infections were determined using logistic regressions.

## 3. Results

### 3.1. Sociodemographic Characteristics of the Respondents in the Study Area

The participants had the following sociodemographic characteristics: majority were females (53.3%), aged 6–9 (54.4%), with uneducated mothers (65.0%) and fathers (62.9%), and educated at Nora Mender school (75.2%) (Table 2).

### 3.2. The Prevalence of STH Infections Among Participants Based on Sociodemographic Characteristics

The prevalence and distribution of participants based on age, sex, school names, mothers' education, and fathers' education showed that most students were infected in the age group of 10–14 (33.6%), males (28.2%), with uneducated mothers and with uneducated fathers (35.5%), and educated at Alemmia school (27.8%) for these infections. However, *A. lumbricoides* infection was higher in Alemmia school (15.7%), while hookworm infection was higher in Nora Mender school (13.8%). The overall prevalence of *A. lumbricoides* and hookworm was higher in Alemmia school (27.8%) than in Nora Mender school (24.4%) (Table 3). The combined prevalence of the two parasites was 25.3%.

### 3.3. Prevalence of Parasitic Infection Among Respondents

Out of 463 stool specimens collected and examined, 129 (27.9%) were found positive for one helminth infection. Four species: *A. lumbricoides*, hookworm, *Hymenolepis nana*, and *Schistosoma mansoni* of parasitic infections were identified in the study. Hookworm was the first predominate parasite detected in 62 (13.4%) followed by *A. lumbricoides* 55 (11.9%). Additionally, *H. nana* and *S. mansoni* were also detected in eight (1.7%) and four (0.8%), respectively. Males (68; 14.7%) were mostly affected than females (61; 13.2%) (Table 4).

### 3.4. Intensity of Infections in the Study Area

In the study area, the intensity of *A. lumbricoides* and hookworm infections was classified as light (Table 5).

### 3.5. Strength of Association Between Risk Factors and A. lumbricoides and Hookworm Infections

#### 3.5.1. Multivariate Logistic Regression

Based on the multivariate logistic regression analysis, uneducated mothers, uneducated fathers, not using latrine/toilet at home, not using latrine/toilet at school, irregular wearing of shoes, untrimmed fingernail status, the absence of handwashing facility at home, not washing hands after using the toilet, and not washing hands before meal were predictor factors for STH infection in the study area (*p* < 0.05) (Table 5).

Students having uneducated mothers were 24.99 times (AOR = 24.99; 95% CI = 7.05–88.67; *p* < 0.001) more likely to contract STH infections than those having educated mothers. Students having uneducated fathers were 3.03 times (AOR = 3.03; 95% CI = 1.18–7.77; *p* = 0.021) more likely to contract STH infections than those having educated fathers. Students not using latrine/toilet at school were 4.53 times (AOR = 4.53; 95% CI = 1.89–10.85; *p* < 0.001) more likely to contract STH infections than those using latrine/toilet at school.

Students that are irregular in wearing shoes were 18.59 times (AOR = 18.59; 95% CI = 6.89–50.13; *p* < 0.001) more likely to contract STH infections than those who always wear shoes. Students having untrimmed fingernail status were 7.31 times (AOR = 7.31; 95% CI = 2.57–20.81; *p* < 0.001) more likely to contract STH infections than those having trimmed fingernails. Students having no handwashing facility at home were 5.86 times (AOR = 5.86; 95% CI = 2.19–15.64; *p* < 0.001) more likely to contract STH infections than those having a handwashing facility at home. Students that are not washing hands after using the toilet were 14.87 times (AOR = 14.87; 95% CI = 3.97–55.75; *p* < 0.001) more likely to contract STH infections than those who wash hands after using the toilet. Students that were not washing hands before a meal were 30.05 times (AOR = 30.05; 95% CI = 11.05–81.74; *p* < 0.001) more likely to contract STH infections than those washing hands before a meal (Table 6).

## 4. Discussion

In the current study, the overall prevalence of *A. lumbricoides* and hookworm infections among students attending two primary schools was found to be 25.3% (95% CI: 21.5%–29.4%). According to the WHO classification, areas of infection risk are categorized into three groups: high-risk areas with a prevalence greater than 50%, moderate-risk areas with a prevalence between 20% and 50%, and low-risk areas with a prevalence lower than 20% [21]. Consequently, the study area in question falls within the moderate-risk category, indicating that no intervention is required at present but rather a case-by-case treatment approach.

The prevalence in the present study is higher than in the previous studies from Handuras (18.9%) [22], Northeast India (16.8%) [23], Adamawa state in Nigeria (19.4%) [24], Ngorongoro Conservation Area in Tanzania (17%) [25], Ethiopia (21.6%) [17], Sekela, Western Ethiopia (16.1%) [26], Gedeo zone (22.2%) [27], and Birbir town (14.2%) [28]. However, it is lower than in previous studies from Kandahar in Afghanistan (26.2%) [29], Laguna de Perlas (Nicaragua) (39.8%) [30], Ethiopia (27.5%) [31], southern Ethiopia (41.1%) [32], Amhara region (37.4%) [33], Tachgayint woreda (31.7%) [34], Yeki district (27.5%) [35], Kola Diba (42.7%) [9], Mettu town (67.1%) [36], and in wetland and nonwetland area of Blue Nile Basin (30.3%) [19]. The differences in study periods, the location of the study area, personal hygiene, and factors relating to environmental hygiene, ecological factors, as well as economic and cultural issues affecting students and the community may all be contributing causes to these variations in prevalence [32, 37]. The sociocultural determinants, behavioral traits, climatic conditions, application of preventive and control measures, and frequency and application of mass drug administration on intestinal parasites among different countries and regions may all play a role in the inconsistent prevalence of the infection.

Ascariasis is a common public health problem of preschool and primary schoolchildren in developing countries [38]. *A. lumbricoides* was 11.9% prevalent in the study area. It was categorized as a low-risk area by the WHO guidelines for classifying infection-risky places. This result was higher than those of studies done in Brazil (2.4%) [39], Bolifamba, Buea in Cameroon (3.9%) [40], Ngorongoro Conservation Area in Tanzania (4%) [25], and Ethiopia (11.2%) [41]. However, it is lower than the results of several earlier investigations reported a higher prevalence of *A. lumbricoides* in Kandahar in Afghanistan (18.7%) [29], Kakamega County in Kenya (43.5%) [42], Ethiopia (13.98%) [43], shore of Lake Hawassa (44.4%) [44], Dessie city (18.5%) [45], Blue Nile Basin in northwest Ethiopia (20.4%) [19], and Gedeo zone (18.6%) [27]. This might be caused by subpar sanitation, a lack of restrooms, the area's elevation, the kind of soil, or the fact that autumnal cases were highest in September [38]. *A. lumbricoides* was found in one type of individual in the other study, which is comparable to the current study. This shows that these parallels may be due to similarities in the physical setting of the study area, ecological factors, as well as sociocultural influences on students and the general public.

The prevalence of hookworm in the study area was 13.4%. According to WHO guidelines to classify the risky areas of infections, it was grouped under low-risk area. This result was lower than that of the study done in the Amhara region (20.6%) [33], Kerewo town (37.4%) [46], Dera district (21.7%) [47], and Durbete (46.9%) [48]. However, the result of the present study was higher than that of the study done in Northeast India (7.4%) [23], Benin (3.2%) [49], Honduras (1.9%) [22], Adamawa state in Nigeria (5.0%) [24], Kandahar in Afghanistan (7.5%) [29], Ngorongoro Conservation Area in Tanzania (13%) [25], Laguna de Perlas (Nicaragua) (12.7%) [30], Bolifamba, Buea in Cameroon (2%) [40], Ethiopia (8.89%) [50], Ethiopia (10.4%) [17], and Ethiopia (12.51%) [43]. This difference among the above findings with the present one might be due to several environmental, social, and geographical factors [51], and might be due to the difference in socioeconomic status, health information, waste disposal system, sample collection season, soil type, as well as climatic and topographic factors of the study areas.

When we look at the prevalence of infections in each school, the highest prevalence (26.1%) was found in the students of Alemmia primary school, while the lowest prevalence (25.1%) was found in the students of Nora Mender primary school. Health education seeks to modify people's attitudes toward their health and cleanliness as well as their behavior in general. Knowledge is frequently increased by disseminating information on the disease and the potential adoption of preventive actions. This may be due to the fact that Nora Mender primary school and the health facility are relatively close by (side by side), making it easier for health professionals to convey information to the schoolchildren about the healthcare system than at Alemmia primary school, which is located far away from the health center. Based on parasite infection, Alemmia primary school was highly prevalent in *A. lumbricoides* compared to Nora Mender, and in hookworm, Nora Mender primary school was highly prevalent compared to Alemmia primary school. This might be the position of the environment and their soil type, which means Alemmia primary school is slightly elevated and has loam soil in nature than Nora Mender primary school, so this type of environmental nature is suitable for *A. lumbricoides* [52]. In Nora Mender primary school, the environment has clay soil type and has a lower elevation point than Alemmia primary school, so this type of soil and the elevation point lower than 2000 m above sea level are suitable for hookworm [53].

In this study, the age group from 10 to 14 years old was more affected than 6–9 years. This difference might be due to the fact that these children have a better chance of attending school. In addition, the main reported reasons for not taking treatment were not attending school and treatment not given. These findings can be justified by the high coverage observed in our study among school-attending children [54]. Similarly, the studies done in eastern Côte d'Ivoire [55], Ethiopia [54], Rama town [56], Birbir town [28], and Medebay Zana woreda in Tigray [57] found consistent results. However, the results of the studies done in Northeast India [23], Gog Gob [27] primary schools in northwest Ethiopian [58], and Ogun state in Nigeria [59] were contradictory.

According to WHO guidelines [20], the infection of STHs in this study was classified as light-intensity infections. The intensity of STHs is categorized as light, moderate, or heavy based on the arithmetic mean of positive participants [60]. Almost all positive cases of *A. lumbricoides* and hookworm infection exhibited light intensity, which is consistent with previous reports in the Gurage zone of south-central Ethiopia [34]. However, other previous studies have reported occurrences of moderate- and heavy-intensity infections rather than light-intensity infections [61]. This variation could have a positive impact on validating the elimination of *A. lumbricoides* and hookworm infections and the appearance of infections in different countries, including our own, and the study area may be subject to a routine deworming treatment.

In this study, not washing hands before eating showed a significant association with the prevalence of STH infection. Similar findings were reported in various locations in Ethiopia, including Durbete town, Dessie city, southern Ethiopia, Ambo town, Goro southwest Shewa, and the shore of Lake Hawassa [10, 11, 45, 48, 50, 62]. The WHO states that areas with poor sanitation tend to have the highest prevalence of such infection [63]. Another risk factor that exhibited a statistically significant association with STH infection was the mother's education level. In this study, it was found that the mother's education level was associated with the prevalence of *A. lumbricoides* infection among students. Similar findings have been reported in previous studies conducted in various regions of Ethiopia, such as in southern Ethiopia [43, 50], in Mettu town [36], and in Ambo town [62].

The absence of latrine/toilet usage at school was found to be significantly associated with the development of STH infection in this research. Similar results were reported in studies conducted in Sekela, western Ethiopia [26], and Endemata primary school at Debre Markos town [13]. These consistent findings highlight the importance of proper sanitation facilities, specifically latrine/toilet usage, in reducing the risk of infections.

Untrimmed fingernails were found to be significantly associated with the development of STH infection. Consistent findings have been reported in several studies conducted in Ethiopia such as in southern Ethiopia, in Blue basin in northwest Ethiopia [19], in Hiruy Abaregawi rural Debre Tabor [64], in Mettu town [36], in Goro, southwest Shewa [65], in Ejaji [66], in Gob Gob primary school in northwest Ethiopia [58], and in Sekela, western Ethiopia [26]. These consistent findings highlight the importance of maintaining trimmed fingernails as a preventive measure against *A. lumbricoides* infection. Proper hand hygiene, including regular nail trimming, can significantly reduce the risk of transmitting pathogens and subsequent infection.

Additionally, it was found that students with uneducated fathers had a significant association with STH infection. This finding is consistent with other reports from Ambesamie [67], Dessie city [45], Ambo town [62], and Chencha town [68]. And the absence of handwashing facilities at home was also significantly associated with parasite infection in this study. Consistent results supporting this association were reported in Chencha town [68]. These findings highlight the importance of education and access to proper hygiene facilities in preventing and controlling parasite infections. The educational level of the father can influence household practices and awareness regarding hygiene, which in turn can impact the transmission of these parasitic infections. Similarly, the availability of handwashing facilities at home plays a crucial role in promoting hand hygiene and reducing the risk of infection transmission. Therefore, efforts to improve education and hygiene infrastructure can contribute to the prevention and control of STH infection among students.

Not washing hands after using the toilet has shown a significant association with STH infections. Consistent findings supporting this association have been reported in various studies such as in Kandahar in Afghanistan [29], in Northeast India [23], in southern Ethiopia [10, 50], in Blue basin in northwest Ethiopia [19], in Medebay Zana woreda in Tigray [57], and in Birbir town [28]. These consistent results highlight the importance of washing hands after using the toilet as a protective measure against infections, particularly infections caused by STH. Proper hand hygiene, including thorough handwashing with soap and water, is crucial in preventing the transmission of pathogens and reducing the risk of infections.

Irregular wearing of shoes has been found to be significantly associated with the development of STH infection. Consistent results supporting this association have been reported in various studies such as in Kandahar in Afghanistan [29], in Bolifamba Buea in Cameroon [40], in southern Ethiopia [43], in northwest Ethiopia [69], in Gena bossa woreda [46], in Sekela in western Ethiopia [26], and in Tachgayint [34]. These consistent findings highlight the importance of regular and proper use of footwear in preventing hookworm infection. Wearing shoes provides a physical barrier between the feet and the environment, reducing the risk of exposure to hookworm infection present in the soil or contaminated surfaces. Therefore, maintaining regular and appropriate use of shoes can contribute to protecting against hookworm infection and promoting overall foot hygiene. The predominant mode of hookworm transmission through skin penetration highlights the significance of proper sanitation and hygiene practices, especially in environments with contaminated soil. Most of the time, hookworm transmission occurs through skin penetration, although there are instances where hookworm parasites can be transmitted to humans through the ingestion of filariform larvae of *An. duodenale* in contaminated food [70]. In the present study, mothers' education was found to be less likely to be associated with hookworm infection. This finding aligns with the results reported in Indonesia [71]. However, it is reassuring to note that in this study, mothers' education did not appear to be a major risk factor for hookworm infection.

These varying findings could be attributed to factors such as the hygienic conditions of latrines, the quality of household stored water, and indicators of malnutrition such as height and weight for age [72]. It is important to consider that the relationship between these variables and the prevalence of infections can be influenced by various contextual factors, including differences in geographical locations, sanitation practices, and individual behaviors.

## 5. Conclusion

In conclusion, the overall prevalence of *A. lumbricoides* and hookworm infections among students attending two primary schools was found to be 25.3%. The study area falls within the moderate-risk category according to the WHO classification. The prevalence of infections differed between the two primary schools, with higher prevalence observed in Alemmia primary school for *A. lumbricoides* (15.7%) and Nora Mender primary school for hookworm (13.8%). Due to their outdoor playing habits, males had a slightly higher prevalence of infections compared to females. More infections affected the age group of 10–14 years old than 6–9 years old.

The result of infection risk factors indicated that uneducated parents, not using latrine or toilet at school, not having a washing facility at home, not washing hands after toilet and before eating, and not trimming fingernails were identified as cardinal risk factors for *A. lumbricoides* infection, and irregular wearing of shoes, age between 10 and 14 years, and not washing hands after toilet use were major risk factors for hookworm infection among the study subjects and contributed to the high prevalence of *A. lumbricoides* and hookworm infections.

### 5.1. Recommendations

It is essential to plan and carry out preventive and control programs, raise health awareness in study areas, and decrease the burden of diseases. Further integrated control measures must be developed, including a safe water supply, washing program, access to toilets, home education initiatives, shoe wearing, and the creation of an environment free from open defecation. Community awareness must also be increased, and student health must be promoted through health education and good personal hygiene. In order to lessen the impact of the diseases, it is crucial to regularly consecutively deworm students, devise and implement prevention and control programs, and promote health awareness in study locations. Additionally, it is advised to administer a double or treble dose rather than a single dose when treating sick persons.

### 5.2. Limitations of the Study

This study is limited to primary schools, which restricts the generalizability of the findings. Including schools from other districts would have provided a more comprehensive assessment of the prevalence of these infections. The lack of availability of data on open defecation and deworming was considered a limitation for this study. Additionally, the absence of polymerase chain reaction (PCR)–based methods prevented the identification of specific hookworm species. In addition, we recognize several limitations in this study. Firstly, due to limited resources and laboratory facilities, we could not perform molecular diagnostic techniques like polymerase chain reaction (PCR) to distinguish between hookworm species. This means that our findings do not identify the exact species involved, which could have offered clearer insights into the epidemiology and informed species-specific control strategies. Secondly, we did not collect data on open defecation practices, an important risk factor for hookworm transmission. Not including this variable may restrict our analysis of related risk factors and could underestimate the impact of sanitation on the observed prevalence. Despite these limitations, the study offers valuable baseline information for public health planning and emphasizes the need for more research that includes better diagnostics and thorough behavioral data.

## Figures and Tables

**Table 1:** Threshold of intensity of infections.

Threshold of intensity of infection (EPG)			
STH species	Light	Moderate	Heavy
*Ascaris lumbricoides*	1–4999	5000–49,999	≥ 50,000
*Trichuris trichiura*	1–999	1000–9999	≥ 10,000
Hookworms	1–1999	2000–3999	≥ 4000

**Table 2:** Sociodemographic characteristics of study participants among students in the Fogera district, northwest Ethiopia, in 2023 (*n* = 463).

Variables	Categories	Frequency	Percentage
Sex	Male	216	46.7
	Female	247	53.3
	Total	463	100.0

Age	6–9	252	54.4
	10–14	211	45.6
	Total	463	100.0

Name of school	Nora Mender	348	75.2
	Alemmia	115	24.8
	Total	463	100.0

Mothers' education	Educated	162	35
	Uneducated	301	65
	Total	463	100.0

Fathers' education	Educated	172	37.1
	Uneducated	291	62.9
	Total	463	100.0

**Table 3:** The prevalence of STH infection among participants based on sociodemographic characteristics.

Variables	Categories	*N* (%)	Parasitic species			
			*A. lumbricoides*		Hookworm	
			Positive	Negative	Positive	Negative
Age	6–9	252 (54.4)	24 (9.6)	228 (90.4)	22 (8.7)	230 (91.7)
	10–14	211 (45.6)	31 (14.7)	180 (85.3)	40 (18.9)	171 (81.1)

Sex	Male	216 (46.7)	28 (13.0)	188 (87.0)	33 (15.3)	183 (84.7)
	Female	247 (53.3)	27 (10.9)	220 (89.1)	29 (11.7)	218 (88.3)

Name of school	Nora Mender	348 (75.2)	37 (10.6)	311 (89.4)	48 (13.8)	300 (86.2)
	Alemmia	115 (24.8)	18 (15.7)	97 (84.3)	14 (12.2)	101 (87.8)

Mothers' education	Educated	162 (35)	3 (1.9)	157 (98.1)	7 (4.4)	153 (95.6)
	Uneducated	301 (65)	52 (17.2)	251 (82.8)	55 (18.2)	248 (81.8)

Fathers' education	Educated	172 (37.1)	10 (5.8)	162 (94.2)	20 (11.6)	152 (88.4)
	Uneducated	291 (62.9)	45 (15.5)	246 (84.5)	42 (14.4)	249 (85.6)

Total		463 (100)	55 (11.9)	408 (88.1)	62 (13.4)	401 (86.6)

**Table 4:** The prevalence of parasitic infection among students in the Fogera district, northwest Ethiopia, in 2023 (*n* = 463).

Study subject (*n* = 463)	*A. lumbricoides* *n* (%)	Hookworm *n* (%)	*S. mansoni* *n* (%)	*H. nana* *n* (%)	Total *n* (%)
Male	28 (13.0)	33 (15.3)	3 (1.4)	4 (1.8)	68 (14.7)
Female	27 (10.9)	29 (11.7)	1 (0.4)	4 (1.6)	61 (13.2)
Total	55 (11.9)	62 (13.4)	4 (0.8)	8 (1.7)	129 (27.9)

**Table 5:** Intensity of infections in the study area.

Types of parasites	Arithmetic mean	Intensity of infections
*Ascaris lumbricoides*	459.5	Light
Hookworm	475.8	Light

**Table 6:** Logistic regression analysis of important risk factors for soil-transmitted helminth infections.

Variables	Categories	Parasite infection status		COR		AOR	
		Positive (*n*, %)	Negative (*n*, %)	95% CI	*p* value	95% CI	*p* value
Sex	Male	60 (12.96)	156 (33.7)	1.28 (0.84, 1.95)	0.246		
	Female	57 (12.3)	190 (41.04)	1			

Grade	5–7	62 (13.4)	168 (36.3)	0.68 (0.14, 3.28)	0.63		
	1–4	53 (11.45)	170 (36.72)	0.85 (0.55, 1.29)	0.44		
	Kindergarten	2 (0.4)	8 (1.73)	1			

Name of school	Nora Mender	87 (25.0)	261 (56.4)	1.06 (0.65, 1.72)	0.82		
	Alemmia	30 (26.08)	85 (18.4)	1			

Age	10–14	71 (15.3)	140 (30.2)	2.27 (1.48, 3.49)	< 0.001^∗^	1.8 (0.72, 4.5)	0.21
	6–9	46 (10.0)	206 (44.5)	1		1	

Mothers' education	Uneducated	108 (23.33)	189 (40.8)	6.69 (3.55, 12.6)	< 0.001^∗^	24.9 (7.05, 88.67)	< 0.001^∗∗^
	Educated	9 (1.94)	157 (33.9)	1		1	

Fathers' education	Uneducated	88 (19.0)	203 (43.8)	2.14 (1.34, 3.43)	0.002^∗^	3.03 (1.18, 7.77)	0.021^∗∗^
	Educated	29 (6.3)	143 (30.9)	1			

Latrine/toilet usage at home	No	95 (20.5)	230 (49.7)	2.18 (1.30, 3.64)	0.003^∗^	0.96 (0.33, 2.80)	0.94
	Yes	22 (47.5)	116 (25.1)	1		1	

Latrine/toilet usage at school	No	82 (17.7)	62 (13.4)	11.45 (6.69, 18.86)	< 0.001^∗^	4.53 (1.89, 10.85)	0.001^∗∗^
	Yes	35 (7.6)	284 (61.4)	1		1	

Shoe-wearing habit	Sometimes	109 (45.1)	72 (15.6)	17.39 (10.15, 29.82)	< 0.001^∗^	18.59 (6.89, 50.13)	< 0.001^∗∗^
	Always	8 (1.7)	274 (59.2)	1			

Drinking water source	Well water	69 (14.9)	101 (21.8)	4.88 (2.84, 8.38)	< 0.001^∗^	2.79 (0.95, 8.22)	0.063
	Stream	26 (5.6)	88 (19.0)	2.11 (1.13, 3.94)	0.019^∗^	1.09 (0.34, 3.54)	0.88
	Tap water	22 (4.8)	157 (33.9)			1	

Status of fingernails	Untrimmed	99 (21.4)	171 (36.9)	5.6 (3.27, 9.71)	< 0.001^∗^	7.31 (2.57, 20.81)	< 0.001^∗∗^
	Trimmed	18 (3.9)	175 (37.8)	1		1	

Handwashing facility at home	No	93 (20.1)	169 (36.5)	4.06 (2.47, 6.66)	< 0.001^∗^	5.86 (2.19, 15.64)	< 0.001^∗∗^
	Yes	24 (5.2)	177 (38.2)	1		1	

Handwash after using toilet	No	108 (23.3)	214 (46.2)	7.4 (3.63, 15.11)	< 0.001^∗^	14.87 (3.97, 55.75)	< 0.001^∗∗^
	Yes	9 (1.9)	132 (28.5)	1		1	

Handwash before feeding	No	92 (19.9)	42 (9.1)	26.6 (15.4, 46.04)	< 0.001^∗^	30.05 (11.05, 81.74)	< 0.001^∗∗^
	Yes	25 (5.4)	304 (65.7)	1		1	

Eating of raw vegetables or fruits	Yes	93 (20.1)	187 (40.4)	3.29 (2.01, 5.41)	< 0.001^∗^	1.65 (0.58, 4.64)	0.35
	No	24 (5.2)	159 (34.3)			1	

*Note:* Edu = Education.

Abbreviations: AOR = adjusted odds ratio, COR = crude odds ratio.

^∗^Indicates COR-associated value and we should do the AOR association.

^∗∗^Indicates AOR-associated factors, meaning the factors are highly related with the prevalence.

## Data Availability

The data that support the findings of this study are available from the corresponding author upon reasonable request.

## Supporting Information

Supporting information related to this paper, including extended data tables, additional figures, and detailed method descriptions, is included to support the findings and is available upon request or in the online supporting file.
