# Supplementary material for: Prevalence and Risk Factors of Soil-Transmitted Helminths Among Students in Fogera District, Northwest Ethiopia
Source: Glob Health Epidemiol Genom. 2025 Oct 31;2025:3259544. doi: 10.1155/ghe3/3259544 (PMC12595231; doi:10.1155/ghe3/3259544)
Supplement: Supporting Information — Additional supporting information can be found online in the Supporting Information section. [file 3259544.f1.docx]

## Questionnaire for the data collection on STHs risk factors (English version)

**General information**

Code no. -------------------

Sample ID------------------

Date-------------------------

Grade -----------------

**General direction**: Please put a **response number** under the column of “**Code**” for the answer.

| **factors in relation to STH infection** | | | | |
| --- | --- | --- | --- | --- |
| **No.** | **Questions** | **Responses** | **Code** | **Remark** |
|  | Sex | 1. Male 2. Female |  |  |
|  | Age | 1. 6-9 2. 10-14 |  |  |
|  | Mother education (Formal education) | 1. Educated   1. Uneducated |  |  |
|  | Father education (Formal education) | 1. Educated 2. Uneducated |  |  |
|  | Availability Latrine/toilet at home | 1. Yes 2. No |  |  |
|  | Latrine/toilet utilization at school | 1. Yes 2. No |  |  |
|  | Shoe wearing habit | 1. Some times 2. Always 3. Never |  |  |
|  | Drinking water source | 1. Tap water 2. Stream/river water 3. Well water |  |  |
|  | Did you trim your finger nail | 1. Trimmed 2. Untrimmed |  |  |
|  | Hand washing facility at home | 1. Yes 2. No |  |  |
|  | Hand washing after toilet | 1. Yes 2. No |  |  |
|  | Hand washing habit before eating | 1. Yes 2. No |  |  |
|  | Eating raw vegetables or fruits | 1. Yes 2. No |  |  |

**Nomenclature**

| AOR | Adjusted Odds Ratio |
| --- | --- |
| CDC | Center for Disease Control and Prevention |
| CI  COR | Confidence Interval  Crude Odds Ratio |
| SGZHD | South Gondar Zone Health Department |
| STHs | Soil Transmitted Helminthes |
| WHO | World Health Organization |
